# When a Discovery Is a Rediscovery: Do We Know the History of Our Own Subject?

**DOI:** 10.1093/function/zqab030

**Published:** 2021-06-08

**Authors:** Ole H Petersen

**Affiliations:** School of Biosciences, Cardiff University, Cardiff, Wales CF10 3AX, UK

It is an old Soviet joke that “The future is certain; it is only the past that is unpredictable,” but history in general is often reinterpreted, and often in the interest of those in power. This process is made easier by the inevitable existence of some sloppy scholarly work that will help unscrupulous “influencers” to spread uncertainty and allow radical misinterpretations. Science is of course supposed to be a rational evidence-based activity and should therefore not have such problems with its own history. However, sloppy referencing, not picked up by sloppy reviewing, does occur. When checking earlier work carefully, it turns out that not all generally accepted narratives concerning when and by whom discoveries were made are correct.

Do we care about the history of our scientific research fields? Unlike those working in other areas of intellectual life, for example, the arts, philosophy, and politics, scientists in general do not seem to worry much about getting the past right. Typically, an original research article refers in its introduction to a review article when describing the scientific background. What is known about the history of a particular field can therefore effectively be determined by a single review article in a prominent journal. Whereas even minute details of recent findings in a field will be tested, confirmed, modified, or rejected, the broad history of a field will often not be revisited and therefore not revised.

My interest in this problem was reawakened when I recently completed a review article for *Physiological Reviews* dealing with the exocrine pancreas. One of the sections in this review deals with Ca^2+^-activated Cl^–^ channels, as these pores play a key role in the control of secretion.^1^ Surveying the general literature on this channel, which is found in many different tissues in the body and has many different functions, I read the following introductory statement in a highly cited review^2^: “Many cell types express a type of Cl^−^ channel that is activated by cytosolic Ca^2+^ concentrations ([Ca^2+^]_i_) in the range of 0.2–5 µM. For example, in *Xenopus* oocytes, where these channels were first described in the early 1980s (1, 2)…” The two references in this introductory statement are to papers published in 1982 and 1983, respectively, in which there are no references to earlier descriptions of this channel. Unfortunately, the statement is misleading. As pointed out in my recent review article,^1^ this channel was discovered by the late Sir Michael Berridge and his colleagues in the early 1970s and most completely described in their 1975 article in the *Journal of Physiology*.^3^ In this article, there is even a clear diagram illustrating how these Cl^–^ channels, activated by a rise in the cytosolic Ca^2+^ concentration, are essential for the process of fluid secretion in insect salivary glands.^3^ The Ca^2+^-activated Cl^–^ channels were extensively characterized in mouse pancreatic acinar cells and described in many papers published several years before their apparent “discovery” in oocytes.^1^ It was shown that acetylcholine opens Cl^–^ channels in the pancreatic acinar cell membrane and that intracellular Ca^2+^ injection can mimic this effect. The anionic selectivity of the Ca^2+^-activated Cl^–^ channel in the acinar membrane was established with the following permeability sequence (in descending order of permeability): NO_3_^–^ > I^–^ > Br^–^ > Cl^–^.^1^ This same sequence was (re)established many years later in studies on the Cl^–^ channel in *Xenopus* oocytes and other cells, but again without reference to the original finding.^2^ The 2005 review article on Ca^2+^-activated Cl^–^ channels^2^ has a short section dealing with fluid secretion by exocrine glands, but even in this section there are no references to the original work carried out in the 1970s. Instead, another review article is cited, in which none of the original papers are mentioned, referring—again—to other review articles. This problematic pattern, seen in many review articles in many journals, is the reason that *Function* insists that discoveries reported in the original research articles are cited in our Evidence Reviews.

The misleading information about the discovery of Ca^2+^-activated Cl^–^ channels^2^ had consequences, as virtually all subsequent investigators and review writers continued to perpetuate the myth that these channels were discovered in oocytes and neurons in the 1980s, disregarding the reports of the much earlier discovery of these channels in the exocrine glands.

Ca^2+^-activated Cl^–^ channels are now known to be TMEM16 proteins,^4^ and there is an extensive literature about their functions, particularly in the nervous system. In 2013, an interesting study reported activation of the TMEM16A channel (also often referred to as ANO1) in nociceptive sensory neurons by local, oscillating, Ca^2+^ signals generated by Ca^2+^ release via IP_3_ receptors.^5^ However, this phenomenon was discovered, and described in detail, many years earlier in a study of pancreatic acinar cells.^6^

The field of Ca^2+^-activated Cl^–^ channels in exocrine glands may be regarded as esoteric by neuroscientists, but may become central in the current COVID era. A very recently published paper shows that the lungs of COVID-19 patients contain pneumocytes with activated SARS-CoV-2 spike protein at the plasma membrane level. These cells have increased cytosolic Ca^2+^ oscillations leading to increased TMEM16 activity, potentially causing increased Cl^–^ secretion, which could lead to alveolar edema.^7^

For most human endeavors, we believe that an accurate knowledge of the past is important, and it is my personal belief that this must also be the case for science and, therefore, of course, also for physiology. If this is so, then we should at least give some priority to getting the history of our many different research fields right. Review writers and referees should therefore not only be concerned about the accuracy of the description of what is currently known in a particular field but also check carefully that the history of the field is correctly described.

As always, rectification of a problem requires in the first instance recognition that there is a problem. The stark example I have given in this editorial is probably not unique and may inspire others to check whether their particular field has an accurate knowledge of its past. I would be interested to hear about other cases where errors, or perhaps even deliberate omissions of key references in a prominent review article, have distorted the general perception of what was shown by whom and when.
